# An Optimized *in situ* Quantification Method of Leaf H_2_O_2_ Unveils Interaction Dynamics of Pathogenic and Beneficial Bacteria in Wheat

**DOI:** 10.3389/fpls.2020.00889

**Published:** 2020-06-23

**Authors:** Pablo Carril, Anabela Bernardes da Silva, Rogério Tenreiro, Cristina Cruz

**Affiliations:** ^1^Plant-Soil Ecology Laboratory, Center for Ecology, Evolution and Environmental Changes (CE3C), Faculty of Sciences, University of Lisbon, Lisbon, Portugal; ^2^BioISI – Biosystems and Integrative Sciences Institute, Faculty of Sciences, University of Lisbon, Lisbon, Portugal

**Keywords:** hydrogen peroxide (H_2_O_2_), biotic interactions, image processing, color deconvolution, 3-3′diaminobenzidine (DAB)

## Abstract

Hydrogen peroxide (H_2_O_2_) functions as an important signaling molecule in plants during biotic interactions. However, the extent to which H_2_O_2_ accumulates during these interactions and its implications in the development of disease symptoms is unclear. In this work, we provide a step-by-step optimized protocol for *in situ* quantification of relative H_2_O_2_ concentrations in wheat leaves infected with the pathogenic bacterium *Pseudomonas syringae* pv. *atrofaciens* (*Psa*), either alone or in the presence of the beneficial bacterium *Herbaspirillum seropedicae* (RAM10). This protocol involved the use of 3-3′diaminobenzidine (DAB) staining method combined with image processing to conduct deconvolution and downstream analysis of the digitalized leaf image. The application of a linear regression model allowed to relate the intensity of the pixels resulting from DAB staining with a given concentration of H_2_O_2_. Decreasing H_2_O_2_ accumulation patterns were detected at increasing distances from the site of pathogen infection, and H_2_O_2_ concentrations were different depending on the bacterial combinations tested. Notably, *Psa*-challenged plants in presence of RAM10 accumulated less H_2_O_2_ in the leaf and showed reduced necrotic symptoms, pointing to a potential role of RAM10 in reducing pathogen-triggered H_2_O_2_ levels in young wheat plants.

## Introduction

Accumulation of reactive oxygen species (ROS) is a common plant response to pathogens, having many and often contrasting functions depending on the plant-pathogen system under study (González-Bosch, 2018). Any type of ROS has specific biochemical characteristics and most of them are extremely unstable (Mittler, 2017). However, hydrogen peroxide (H_2_O_2_) is relatively more stable having a half-life time of more than 1 ms, and is considered the predominant ROS involved in cellular signaling (Černý et al., 2018). ROS regulate numerous immune responses to invading microorganisms, including both the hypersensitive and programmed cell death responses, the cross-linking of cell wall proteins, the deposition of callose or the activation of redox-sensitive genes. Furthermore, ROS, participate in cell-to cell signal transduction to systemic tissues, where localized ROS bursts can induce defenses to prepare (or “prime”) plants for future challenges (Torres et al., 2006; Noctor et al., 2018).

Changes in ROS levels also occur during beneficial interactions. Upon contact with plant growth promoting rhizobacteria (PGPR), plant H_2_O_2_ levels often increase, and H_2_O_2_ accumulation can be primed for enhanced resistance against pathogens (Ahn et al., 2007). Notably, PGPR can alleviate oxidative stress by modifying the activity of antioxidant enzymes and by modulating H_2_O_2_ concentrations in the leaf (Lucas et al., 2014; García-Cristobal et al., 2015; Singh and Jha, 2017). As a consequence, PGPR have emerged as a promising alternative to increase oxidative stress tolerance and disease resistance in plants (Islam et al., 2014; Pieterse et al., 2014). Wheat (*Triticum aestivum*) is challenged by several bacterial pathogens, which can cause severe diseases and pests (Valencia-Botín and Cisneros-López, 2012). The pathogen *Pseudomonas syringae* pv. *atrofaciens* (*Psa*) can infect wheat leaves and cause longitudinal brown necrotic-like lesions in the in the site of pathogen entrance resembling those occurring during oxidative stress as a result of high accumulation of ROS in the infection point (Duveiller, 1997).

Despite oxidative stress and pathogen responses are well-studied processes involving H_2_O_2_ in various ways, it is unclear how H_2_O_2_ signaling operates in the presence of both pathogenic and beneficial bacteria. This study aims to provide an optimized protocol for *in situ* detection and quantification of relative H_2_O_2_ concentrations in wheat leaves bacterized with pathogenic and beneficial bacteria, both individually or in combination. This was achieved by combining the 3-3′diaminobenzidine (DAB) staining method previously used for plant material (Wang et al., 2007) and image processing with Fiji/ImageJ software. The combination of these techniques enabled the application of a linear regression model correlating the intensity of the pixels resulting from DAB staining with a given concentration of H_2_O_2_. This model was suitable for detection and quantification of relative H_2_O_2_ accumulation in different leaf areas upon infiltration with *Psa* and root-inoculation with the PGPR *Herbaspirillum seropedicae* strain RAM10, either individually or in combination. Furthermore, the area of the lesion caused by *Psa* was measured in presence or absence of previous root-inoculation with RAM10.

This method was suitable to analyze and compare the differential H_2_O_2_ induction effect between the experimental conditions tested. Our results show that H_2_O_2_ accumulates at different degrees depending on the leaf region or the different plant-bacteria interactions. Notably, *Psa*-challenged plants in presence of RAM10 showed reduced H_2_O_2_ accumulation as well as less necrotic symptoms in the leaf, suggesting a PGPR-mediated reduction in oxidative stress levels upon pathogen challenge.

## Materials and Methods

### Bacterial Growth Conditions

*Herbaspirillum seropedicae* strain RAM10 (RAM10), isolated from *Graminaceae* plants (Olivares et al., 1996), was grown in DYGS medium (composition g L^–1^: 2.0 glucose; 2.0 malic acid; 2.0 yeast extract; 1.5 peptone; 0.5 K_2_HPO_4_; 0.5 MgSO_4_ 7H_2_O; 1.5 L-glutamic acid; pH 6.5) at 28°C and 120 rpm, under dark conditions overnight. Bacterial cells collected by centrifugation (2374 × *g*, 10 min) were washed twice with sterile deionized water (SDW) and resuspended in 1/4 Hoagland solution (Hoagland and Arnon, 1950) to a final OD_600_ = 1 (10^9^ CFU/mL) for root inoculation of seedlings.

*Pseudomonas syringae* pv. *atrofaciens* strain 2213 (*Psa*), isolated from *T. aestivum* plants (McCulloch, 1920) was grown in NB medium (composition g L^–1^: 10.0 tryptone; 5.0 meat extract; 5.0 NaCl) at 28°C and 120 rpm, under dark conditions overnight. Bacterial cells collected by centrifugation (2374 × *g*, 10 min) were washed twice and resuspended in SDW to a final density of 10^9^ CFU/mL for pressure infiltration into the leaves.

### Plant Growth Conditions

Wheat (*Triticum aestivum* cultivar “Trigo mole”) seeds were surface-sterilized (1.5 min 70% ethanol; 1× wash in SDW; 3 min NaOCl; 10× wash in SDW), soaked for 12 h in SDW and heat-treated (10 min, 50°C; 1 mL/seed). Seeds were then aseptically transferred to square Petri dishes (20 seeds per dish) containing 1.5% water agar and incubated at 30°C in dark conditions for 48 h and kept in a growing chamber with a 16/8 h light/dark photoperiod, temperature 25/20°C and relative humidity (RH) 70%/50%), for 48 h. Four day-old seedlings were then transferred to empty tip boxes containing 225 mL of 1/4 Hoagland solution, with the leaves emerging from the holes of the rack and the Hoagland solution bathing the roots.

### Measurement of Leaf Symptoms

Four day-old seedlings were divided in four groups (four tip boxes) composed of 7 seedlings each, with three replicates per group: control, non-bacterized (C); RAM10-inoculated (RAM10); *Psa*-infiltrated (*Psa*) and both RAM10-inoculated and *Psa-*infiltrated (RAM10 + *Psa*). In both RAM10 and RAM10 + *Psa* groups, 25 mL of RAM10 suspension was added to the Hoagland solution bathing the roots to a final density of 10^8^ CFU/mL (250 mL final Hoagland volume). Each box was then sealed in plastic gas exchange bags. Four days after RAM10 inoculation, *Psa* and RAM10 + *Psa* groups were pressure infiltrated in the central part of the leaf with 1 mL of *Psa* culture using a needless syringe. Leaves of C and RAM10 groups were pressure infiltrated with 1 mL of SDW. Five days after infiltration, leaves were cut, mounted in transparent plastic slides and pictures were taken. The area of both necrotic and chlorotic symptoms was quantified from the digitalized images using the image processing software Fiji/ImageJ (ImageJ, RRID:SCR_003070). For this, the affected area was manually defined using both the “polygon” selection tool and the “brush” tool to adjust the size of the selection to the shape of the affected area. The size of the affected area was expressed as mm^2^ of both necrotic and chlorotic symptoms.

### Construction of DAB-H_2_O_2_ Calibration Curve: Step-by-Step Protocol

(1) Prepare several H_2_O_2_ dilutions ≤ 47 mM from stock solution at 30% (w/w), that is 9.8 M, with ultra-pure water or sterile deionized water (SDW).
  • Note: H_2_O_2_ Molar mass = 34.01468 g mol^–1^, density = 1.11 g mL^–1^.
(2) Measure the absorbance of the H_2_O_2_ dilutions at 240 nm in a quartz cuvette, after adjusting zero absorbance with the water used for dilutions.
(3) Calculate the exact H_2_O_2_ concentration of the different solutions using Lambert-Beer law, considering the molar attenuation coefficient or absorptivity (ε) for H_2_O_2_ at 240 nm equal to 42.3 M^–1^ cm^–1^ and pathlength (l) = 1 cm.
  Note: Lambert-Beer law is valid up to an absorbance ≤2.
(4) Prepare paper filter disks with an area ≤internal area of a 2 mL microtube. Place the disk inside de microtube in horizontal position.
  Impregnate all the disk surfaces with adequate volume of H_2_O_2_ solution, without overloading, and add the DAB solution (1 mg/mL). Make this in triplicate for each [H_2_O_2_]. Additionally, place 3 disks with DAB only for later background subtraction.
  Note: It is important to avoid overloading of paper filter disks, since the precipitated formed by H_2_O_2_ reaction with DAB may sediment in the bottom of the microtube, underestimating [H_2_O_2_] and subsequent analysis. We used filter disks with a diameter of 55 mm, 16.6 μL of H_2_O_2_ solution and 150 μL DAB per disk.
(5) Incubate the microtubes at room temperature and in dark conditions, overnight.
(6) Take out the disks with clean tweezers and mount the disks in a transparent plastic slide.
(7) Digitalize the disks with a scanner and open the image with Fiji/ImageJ software.
  • Apply the color deconvolution plugin in order to unmix the color vectors of the digitalized disks. From the resulting panel containing DAB color only, select each disk (region of interest, ROI) using the “oval” selection tool and measure the initial average DAB pixel intensity (*ii*), expressed as:
  • $i\,i=\frac{\sum \text{vpx}}{{\text{n}}^{\circ }\,\text{px}}$
  • where *ii* is the initial average pixel intensity; ∑vpx is the sum of the values of the pixels composing the selected area; and n°px is the number of pixels composing the selected area.
(8) Invert the initial average pixel intensity values by using the formula:
  • *iinv*=255-*ii*
  • being *iinv* the inverted average pixel intensity. Note that, for 8-bit images, *i* ranges from 0 (zero = deep brown, highest expression), to 255 (total white).
(9) Subtract the background DAB intensity to the *iinv* values, according to the formula:
  • i⁢f⁢i⁢n⁢a⁢l=i⁢i⁢n⁢v-i⁢b⁢l⁢a⁢n⁢k
  • where *ifinal* is the final intensity of the disk and *iblank* is the average intensity value of 3 filters drenched with DAB only.
(10) Construct a calibration curve correlating the *ifinal* values with the corresponding H_2_O_2_ concentration (μmol H_2_O_2/_cm^2^). Calibration curves with average values (based on triplicates) are presented in Figure 2A.
(11) Quantify average pixel intensity also in the complimentary image and represent in a graph the values of average pixel intensity with the corresponding H_2_O_2_ concentration (μmol H_2_O_2/_cm^2^) (Supplementary Figures 1A,B).

### Quantification of Relative H_2_O_2_ Concentration in Leaves Using 3,3-Diaminobenzidine (DAB)

Ten seedlings from each of the four treatments (C, *Psa*, RAM10 + *Psa*) were grown to detect H_2_O_2_ accumulation in the 1st leaf. Detection of H_2_O_2_ in leaves was carried out using the 3,3-diaminobenzidine (DAB) staining method already used for barley and wheat plants (Thordal-Christensen et al., 1997; Wang et al., 2007), with slight modifications: 1, 2, 6, 24, and 48 h post-inoculation (hpi), leaves were cut and the cut ends were immersed in 1 mL of a solution containing 1 mg/mL DAB dissolved in HCl-acidified (pH 3.8) SDW. Leaves were incubated in a growing chamber overnight to allow DAB uptake and reaction with H_2_O_2_. Solutions were kept under dark conditions.

After incubation, leaves were decolorized in boiling (∼78°C) 95% ethanol for 20 min and transferred into a solution containing water and 20% glycerol.

Leaf segments were placed in filter paper to remove the excess of glycerol solution, mounted in transparent plastic slides, scanned (Epson XP-235) and the images opened with Fiji/ImageJ software. Initial settings of the software were applied to measure area (mm^2^) and mean pixel intensity. Global scale of the image analysis was set as 46.5 pixels = 1 mm. Then, the image was submitted to the plug-in “color deconvolution” using the built-in vector HDAB in order to limit to the DAB dye image. Three different areas (regions of interest, ROIs) were selected for analysis in the DAB only image: from 0 to 4, from 4 to 8 and from 8 to 12 mm from the *Psa* infiltration site. Selection of ROIs was performed using the “rectangle” selection tool. Once the three areas were selected, the “brush” tool was used to adjust the size of the rectangle according to the leaf shape. Then, the area in mm^2^ and the mean intensity of DAB was calculated. Intensity values ranged from 0 (deep brown) to 255 (total white).

The average DAB intensity was calculated according to the formula: i_DAB_ = 255-i, being i_DAB_ = final DAB intensity of the ROI compared to average intensity of total white of the ROI, i = the mean DAB intensity of the ROI. In order to subtract the background of the leaf tissue, the average intensity of 20 leaves pressure-infiltrated with water, incubated in water without DAB, and then destained (blank leaves) was measured and subtracted to the i_DAB_ value calculated for each ROI, according to the formula: f_DAB_ = i_DAB_- i_blank_, being f_DAB_ = final DAB intensity and i_blank_ the average intensity of the blank leaves.

## Results

### *Psa-*Triggered Disease Symptoms in Absence or Presence of RAM10

**FIGURE 1:**
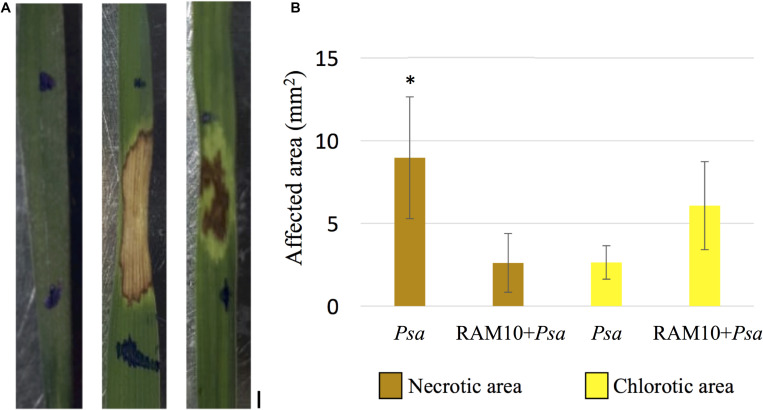
**(A)** Disease symptoms in *Psa* (center) and RAM10 + *Psa* plants (right) 24 h post-infiltration of *Psa* in the center of the 1st leaf; control plant (C) is shown at left; scale bar = 1 mm. **(B)** Necrotic and chlorotic areas composing the affected area in *Psa* and RAM10 + *Psa* plants (mean ± 95% CI, corrected for multiple comparisons; asterisk indicates significant differences in necrotic area between *Psa* and RAM10 + *Psa* leaves).

Infiltration with *Psa* in wheat leaves caused the development of dried brown, necrotic lesions surrounded by chlorosis after 24 h both in *Psa* and RAM10 + *Psa* seedlings (Figure 1A). Despite both diseased leaf areas being similar in size, the proportion of chlorotic and necrotic symptoms was different between the two treatments (Figure 1B). Around 75% of the diseased leaf area in *Psa* leaves was composed of necrotic tissue, which appeared in form of a dried brown area, presumably as a result of the onset a hypersensitive response at the site of pathogen entrance. This necrotic area often reached the borders of the leaf and was surrounded by a thin layer of chlorotic symptoms. Average necrotic area in RAM10 + *Psa* leaves was significantly reduced compared to *Psa* ones, with 30% of the total diseased leaf area showing necrosis, and with chlorosis representing most of the total diseased area.

### Regression Model for H_2_O_2_ Quantification

**FIGURE 2:**
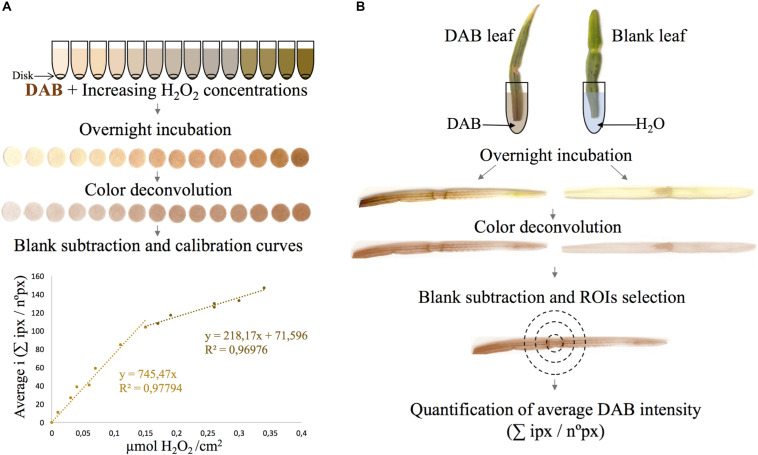
**(A)** Steps for the generation of H_2_O_2_-DAB intensity calibration curve. Disks are put in microtubes, drenched at different H_2_O_2_ concentrations and 1 mg/mL DAB solution is added. After incubation, disks are digitalized and average DAB intensity in the deconvoluted image is determined. Finally, average DAB intensity values are related with those of the several H_2_O_2_ concentrations (μmol H_2_O_2_/cm^2^). **(B)** Leaf incubation in 1 mg/ml DAB solution and processing of the initial leaf RGB image resulting in the deconvoluted 8-bit DAB image. Leaf background average intensity (blank) was subtracted and ROIs were selected in leaf for quantification of the final DAB average pixel intensity of specific ROIs.

A linear regression model to quantify H_2_O_2_ was applied by combining the DAB staining method with image processing using Fiji/ImageJ software. This was done by relating average DAB intensity values to a given amount of H_2_O_2_. A DAB color gradient was generated by incubating filter disks in separate microtubes containing DAB + increasing H_2_O_2_ concentrations. Disks ranged from light to dark-brown stained disks, relative to low to high H_2_O_2_ concentrations (or low to high intensities), respectively (Figure 2A). Disks were digitalized and subjected to the color deconvolution plugin, allowing the separation of the initial RGB image into three 8-bit images, which corresponded to the three vector colors composing the image, being: (1) the brown vector, used for subsequent H_2_O_2_ quantification; (2) the blue vector, not present in the DAB stained disk and (3) a residual channel, also referred to as the complimentary vector, containing the complementary of the other color(s).

Two calibration curves were constructed relating the average DAB intensity values obtained in the DAB only stained section with the corresponding H_2_O_2_ concentration (μmol H_2_O_2_/cm^2^) applied. The first curve ranged from 0 to 104 DAB intensity values and the second one from 104 to 147 values. Both curves showed a linear relationship between the two variables (*R*^2^ ≥ 0.97; Figure 2A).

Previous authors have stressed the importance of taking into account the intensity values of the pixels in the complimentary image, since they may contain shades of DAB, leading to false positive stain separation (Ruifrok and Johnston, 2001; Varghese et al., 2014). In order to correct the intensity values in the DAB only vector, the pixel intensity of the same disks in the complimentary vector was quantified (Supplementary Figure 1A), and a curve relating the average green intensity values versus μmol H_2_O_2_/cm^2^ was plotted (Supplementary Figure 1B). However, contrary to the DAB only vector, the average intensity values of each disk were not proportional to the applied H_2_O_2_ concentration. In fact, several filters with higher H_2_O_2_ concentrations were less stained compared to those drenched with DAB solution only, indicating that average intensity in the complimentary image did not depend on H_2_O_2_ concentration. Because summing these values would decrease the accuracy of H_2_O_2_ quantification, average intensity values in the complimentary image were not considered.

### Determination of H_2_O_2_ Accumulation in Leaves

**FIGURE 3:**
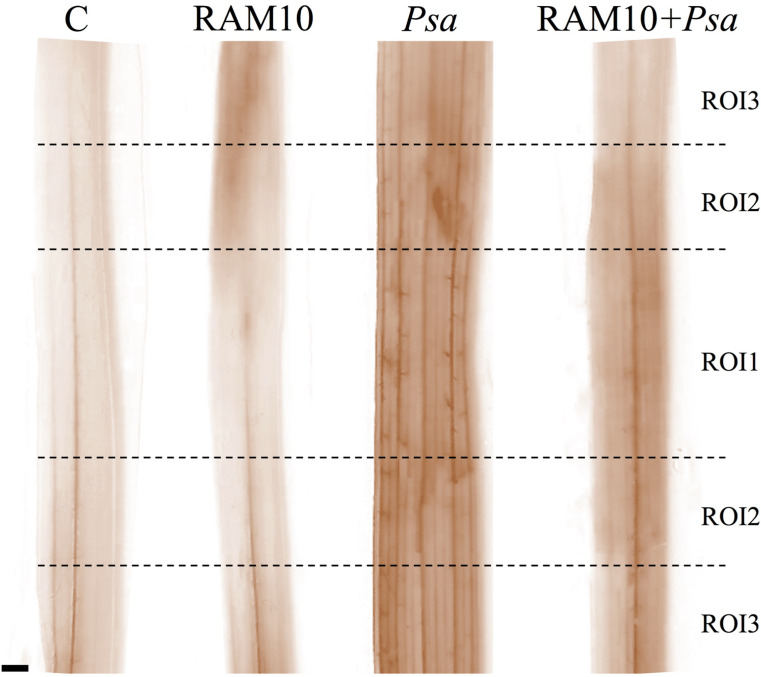
Digitalized DAB-treated leaves 3 h post-*Psa* inoculation and after application of the color deconvolution plugin. These images were used for subsequent ROI selection, measure of average DAB intensity and H_2_O_2_ quantification through the calibration curve appropriate to the range of DAB intensity values; scale bar = 1 mm.

In parallel, wheat leaves were incubated in the DAB solution, digitalized and subjected to the color deconvolution plugin (Figure 2B). DAB color was visualized, and different ROIs were selected to measure the average DAB intensity and determine relative H_2_O_2_ concentration using the calibration curve. Accumulation of H_2_O_2_ was visualized as dark-brown precipitates resulting from the oxidation of DAB by H_2_O_2_ present in the leaf. A more intense staining was observed in the vascular beams (Figure 3).

**FIGURE 4:**
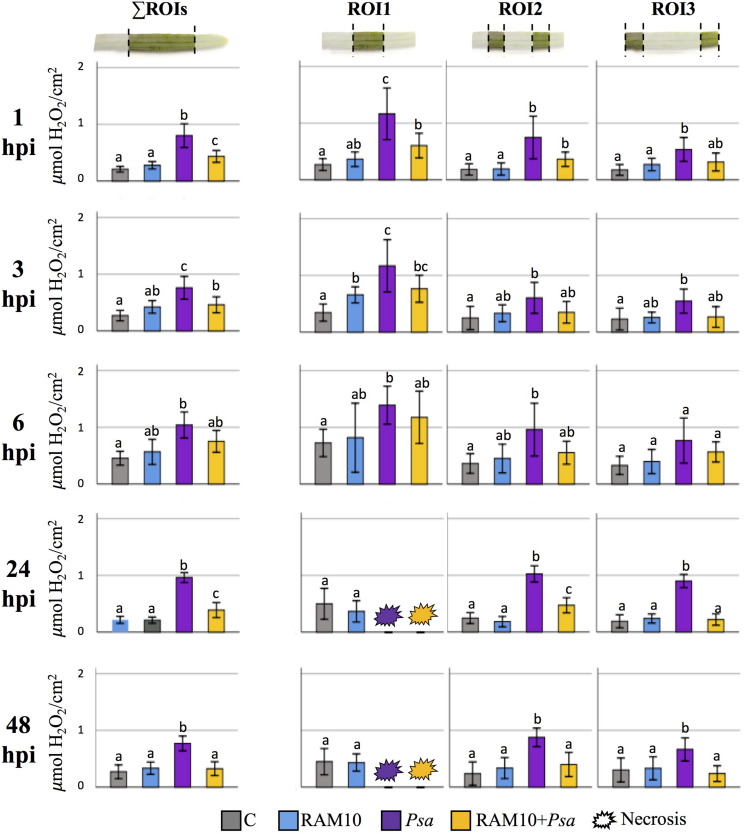
Relative H_2_O_2_ concentration in the entire selected area (∑ROIs) in C, RAM10, *Psa* and RAM10 + *Psa* plants from 1 to 48 h post *Psa* inoculation. The three graphs next to each ∑ROIs represent H_2_O_2_ concentration at increasing distances from the inoculation point (ROI1, ROI2 and ROI3) in C, RAM10, *Psa* and RAM10 + *Psa* plants from 1 to 48 h post *Psa* infiltration. Different letters indicate statistically significant differences between groups (mean ± 95% CI, corrected for multiple comparisons; Games-Howell non-parametric test, *p* < 0.05).

Infiltration of *Psa* induced an active production of H_2_O_2_ in the leaf, both upwards and downwards from the site of infection. Pathogen-infiltrated treatments (*Psa* and RAM10 + *Psa*) showed increased H_2_O_2_ accumulation in the whole selected leaf area (∑ROIs, Figure 4) compared to both C and RAM10 ones. This increase was always more pronounced in *Psa* treatment, compared to which RAM10 + *Psa* plants accumulated significantly less H_2_O_2_. RAM10 treatments showed similar H_2_O_2_ accumulation relative to C ones, indicating that root inoculation of RAM10 did not have a significant H_2_O_2_ induction effect in aboveground tissues.

The analysis of each independent ROI (Figure 4) showed that H_2_O_2_ is produced at different degrees in the leaf, decreasing its accumulation at increasing distances from the infiltration point. After 6 h of *Psa* inoculation, both challenged treatments reached maximum H_2_O_2_ accumulation in ROI1 (1.39 and 1.18 μmol/cm^2^ H_2_O_2_ in *Psa* and RAM10 + *Psa* plants, respectively), which was covered by dried-brown necrotic tissue 24 hpi. Also, in ROI2 and ROI3, H_2_O_2_ levels were always higher in *Psa* plants compared to RAM10 + *Psa* ones, showing significant differences at 24 and 48 hpi.

**FIGURE 5:**
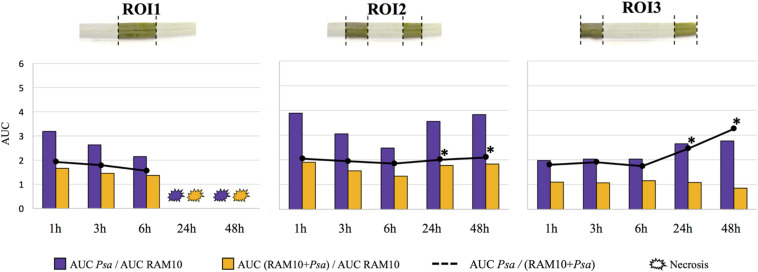
Evolution of cumulative H_2_O_2_ fold induction triggered by *P. syringae* pv. *atrofaciens* in leaves of *Psa* and RAM10 + *Psa* plants relatively to RAM10 ones (bars), expressed as the ratio between the respective area under curves (AUC) at each time post-infection. The degree of RAM10-mediated alleviation of H_2_O_2_ accumulation in leaf tissue, calculated as the AUC ratio between *Psa*-triggered H_2_O_2_ production in *Psa* plants relatively to RAM10 + *Psa* ones, is represented by black lines. Asterisks show statistically significant differences (*p* < 0.05) of the ratio AUC(*Psa*)/AUC(RAM10 + *Psa*).

In order to assess the RAM10-mediated alleviation effect, the area under curve (AUC) of H_2_O_2_ accumulation was calculated at each time post-infection in the different treatments, and the evolution of cumulative H_2_O_2_ fold induction triggered by *Psa* was analyzed for the AUC ratios *Psa*/RAM10 and (RAM10 + *Psa*)/RAM10 (i.e., the H_2_O_2_ induction effect of *Psa* in presence or absence of RAM10 relative to RAM10-only inoculated seedlings; Figure 5, bars), as well as for the *Psa*/(RAM10 + *Psa*) AUC ratio (i.e., the H_2_O_2_ induction effect of *Psa* in absence of RAM10 relative to RAM10 + *Psa* seedlings; Figure 5, black lines).

*Psa*-triggered H_2_O_2_ induction effect was always reduced when the pathogen was inoculated in presence of RAM10. This RAM10-mediated alleviation of H_2_O_2_ accumulation was evident in all timepoints, where *Psa* plants accumulated between 1.5 and 2-fold more H_2_O_2_ relative to RAM10 + *Psa* ones along timepoints. In ROI3, while H_2_O_2_ accumulation in *Psa* plants increased at 24 and 48 hpi, it remained unchanged in RAM10 + *Psa* ones, highlighting a statistically significant alleviation effect of H_2_O_2_ accumulation in this region at 24 and 48 hpi (black line, Figure 5). These results point that PGPR-inoculated plants may be more sensitive to H_2_O_2_ signaling, not requiring its massive accumulation upon a challenge.

## Discussion

### Detection of H_2_O_2_ by DAB Staining Coupled With Imaging Software Analysis

There exist numerous functions accounted to H_2_O_2_ in response to pathogens. Despite its crucial role in plant metabolism, there is little consensus regarding the amount of H_2_O_2_ dynamics in plants challenged with pathogens and pre-treated with PGPR. This is mainly due to both biological variability and technical inaccuracies during its quantification (Queval et al., 2008). Various techniques can quantify H_2_O_2_ contents in plant tissue extracts, such as those relying on absorbance of oxidized products with altered spectral characteristics (Junglee et al., 2014) or on light emission as fluorescence or luminescence (Miller et al., 2012). Enzymatic assays or the use of metal catalysts of H_2_O_2_-dependent reactions are also widely used, both of which can overcome problems of H_2_O_2_ specificity (Queval et al., 2008; Nagaraja et al., 2009). However, some of the challenges that these techniques face include (1) complex interactions between metals or enzymes that may occur during the extraction rather than in the intact tissue, (2) H_2_O_2_ dilution effects at increasing amount of leaf tissue extracted, which may often mask the real H_2_O_2_ response (Queval et al., 2008), (3) manipulation effects during sample preparation.

Image analysis for *in situ* quantification of DAB staining has the advantage over biochemical assays that it is non-destructive and minimizes the manipulation of plant material. Furthermore, DAB staining method relies on the activity of peroxidases present in the leaf, not requiring addition of external peroxidases, which may be another factor affecting ideal *in vivo* conditions. In this work, we presented an optimized *in situ* detection method using DAB staining coupled to image processing to both detect and quantify H_2_O_2_ in leaves.

DAB stained leaves can be digitalized, opened in Fiji/ImageJ and subjected to the color deconvolution plugin, an algorithm developed by Ruifrok and Johnston (2001), which unmixes the color information of the digitalized leaf. The color deconvolution plugin has been previously used for human tissue microscopy analysis (Lessey and Savaris, 2013; Varghese et al., 2014). In this work, this method was adapted for young wheat leaves and used to detect the spatial distribution of the DAB intensity at increasing distances from the infection site. As a result, an image with DAB only staining is generated, and the average intensity of its pixels can be quantified after the selection of specific ROIs. Having DAB stained leaves digitalized, other ROIs can be defined anytime.

In previous studies using DAB staining, leaf H_2_O_2_ content was estimated as the percentage of dark brown DAB pixels relative to the pixels composing the leaf area. In order to express H_2_O_2_ content in concentration units, these studies relied on parallel spectrophotometric assays for H_2_O_2_ quantification (Luna et al., 2011; Liu et al., 2014; Wu et al., 2019). However, these analyses require the involvement of different plant samples for both the DAB staining and the spectrophotometry, since the DAB signal could not directly correlate with specific H_2_O_2_ concentration units. In the present study, the application of a linear model combining average pixel intensity values with known H_2_O_2_ concentrations allowed to quantify relative H_2_O_2_ concentrations in the leaf according to its DAB staining intensity values. The generation of this curve avoided the manual setting of a maximum and minimum threshold intensities in the images, which can itself be subjective, leading to misinterpretations in tissue sample scoring (Varghese et al., 2014). It was neither necessary to linearize the intensity values to OD values, as indicated by previous articles (Ruifrok and Johnston, 2001; Varghese et al., 2014), since the values of DAB intensity were linearly related with the concentrations of H_2_O_2_ and can therefore be used for extrapolating H_2_O_2_ concentration from DAB intensity values. Furthermore, the H_2_O_2_ dilution effects were minimized by selecting small (8 mm) ROIs in intact (stressed or non-stressed) leaves after image processing. Manipulation of the plant material was almost inexistent, since the only stress applied to the plant was an initial cut at the base of the cotyledon, immediately prior to DAB incubation. Considering this, specific ROIs were selected excluding both the basal part of the leaf and its apex, which, in few cases, started to senesce (data not shown).

### Application of the New Method for Studying Biotic Interactions

Leaves infiltrated with *Psa* accumulated H_2_O_2_ both locally and at further distances from the infection point, where dark brown DAB precipitates were found to be more intense in the vascular beams. Tissue-specific localization of H_2_O_2_ associated with vascular tissues has been previously observed (Ślesak et al., 2007) and is in agreement with previous studies which suggest that vascular bundles can synthesize these ROS signals during stress for rapid autopropagation and induction of systemic stress immunity (Libik-Konieczny et al., 2015; Gaupels et al., 2017).

The method proposed in this study was applicable to analyze and compare the differential H_2_O_2_ induction effect of *Psa* in the presence or absence of the PGPR RAM10. H_2_O_2_ accumulated at higher levels in the site of pathogen entrance (ROI1), which became necrotic 24 hpi. These observations suggest that induction of hypersensitive cell death by *Psa* in the site of infection is temporally preceded by H_2_O_2_ accumulation in the site of pathogen entry, while H_2_O_2_ accumulation, but not cell death, was induced in the tissue adjacent to the infiltration point. Since ROS participate in cell-to cell signal transduction to systemic tissues, this ROS accumulation in distant parts from the pathogen entry could be a source of signals for establishment of further defenses to prepare (or “prime”) plants for future challenges (Noctor et al., 2018).

Interestingly, RAM10-treated plants showed consistently less H_2_O_2_ accumulation, where the most remarkable alleviation effect was observed 24 and 48 hpi in the most distal area (ROI3), which maintained a low, initial *Psa*-induced H_2_O_2_ accumulation overtime. These observations suggest that necrosis and H_2_O_2_ signal propagation occurs in both *Psa* and RAM10 + *Psa* plants, but RAM10-inoculated plants can alleviate the degree of H_2_O_2_ accumulation upon *Psa* challenge and maintain the basal levels of stress initially triggered by *Psa* in more distal parts of the leaf, without undergoing a further H_2_O_2_ accumulation. This reduction in ROS levels in challenged plants pre-inoculated with a beneficial microorganism, including bacteria and fungi, has been previously observed. For example, endophytic bacteria-primed *Abelmoschus esculentus* plants expressed lower level of H_2_O_2_ accumulation upon *Sclerotium rolfsii* challenge, compared to unprimed plants, probably due to the enhanced expression of antioxidant enzymes (Ray et al., 2016). In tobacco leaves, *Bacillus atrophaeus* HAB-5 inhibited ROS accumulation in leaves during TMV infection, which was related with enhanced resistance against the virus and inhibition of cell death (Rajaofera et al., 2020). Besides, the fungus *T. harzianum*–mediated biocontrol may be related to alleviating *Rhizoctonia solani*-induced oxidative stress by reducing the levels of hydroxyl radical, O_2_^⋅–^ and H_2_O_2_ after pathogen challenge (Singh and Singh, 2011). Furthermore, the pre-treatment of alfalfa plants with lipopolysaccharides from *Sinorhizobium meliloti* suppressed the yeast elicitor-induced oxidative burst reaction (Albus et al., 2001). One hypothesis to explain this alleviation effect is that RAM10 inoculation may induce weak and transient defense-associated changes in ROS signaling upon contact with roots and this signal may be transmitted to aboveground parts of the plant. Contact with RAM10 could pre-activate H_2_O_2_ signaling in aboveground tissues, avoiding its massive accumulation upon pathogen challenge and increasing plant sensitivity to H_2_O_2_ signaling. Contrarily, without being alerted by a previous contact with RAM10, pathogen infiltration in *Psa* plants would lead to a massive, uncontrolled accumulation of H_2_O_2_, resulting in cellular damage and increased necrotic area (Van Breusegem and Dat, 2006). In relation to this, RAM10 could prime plants to increase antioxidant enzyme activity/production upon a future infection.

## Conclusion

In this work, we report for the first time an integrated protocol that simultaneously allows to detect DAB distribution, to quantify amount of DAB signal in different leaf regions and to relate this signal to a given concentration of H_2_O_2_. The method is non-expensive and applicable to analyze and compare the differential H_2_O_2_ induction effects of wheat plants bacterized with both pathogenic and beneficial bacteria.

This methodology allowed to show that the pathogen *Psa* clearly increased H_2_O_2_ accumulation in infiltrated leaves. On the contrary, both H_2_O_2_ levels and disease symptoms induced by this pathogen decreased in presence of RAM10, suggesting a role for this PGPR in the alleviation of pathogen-induced oxidative stress and the progression of necrotic symptoms.

## Data Availability Statement

The raw data supporting the conclusions of this article will be made available by the authors, without undue reservation.

## Author Contributions

PC and AS designed the experiments and analyzed the data in the H_2_O_2_ quantification part. PC, RT, and CC designed the experiments and analyzed the data concerning the biotic interaction part. PC performed the experiments. All researchers contributed to the research and approved the final version of the manuscript.

## Conflict of Interest

The authors declare that the research was conducted in the absence of any commercial or financial relationships that could be construed as a potential conflict of interest.

## Supplementary Material

FIGURE S1 **(A)** Values of H_2_O_2_ concentration in the disks quantified in the complementary image, their average pixel intensity values (averages of three disk replicates) and aspect of the filter disks. **(B)** Relationship between average pixel intensity detected in the complimentary image and (±STDEV) and the amount of H_2_O_2_ per cm^2^ of disk.

## Abbreviations

AUC: area under curve
DAB: 3,3′-diaminobenzidine
*Psa*: *Pseudomonas syringae* pv. *atrofaciens* strain 2213
RAM10: *Herbaspirillum seropedicae* strain RAM10
ROI: region of interest
SDW: sterile deionized water.
