# Supplementary figures and images for: An Optimized in situ Quantification Method of Leaf H2O2 Unveils Interaction Dynamics of Pathogenic and Beneficial Bacteria in Wheat
Source: Front Plant Sci. 2020 Jun 23;11:889. doi: 10.3389/fpls.2020.00889 (PMC7344315; doi:10.3389/fpls.2020.00889)

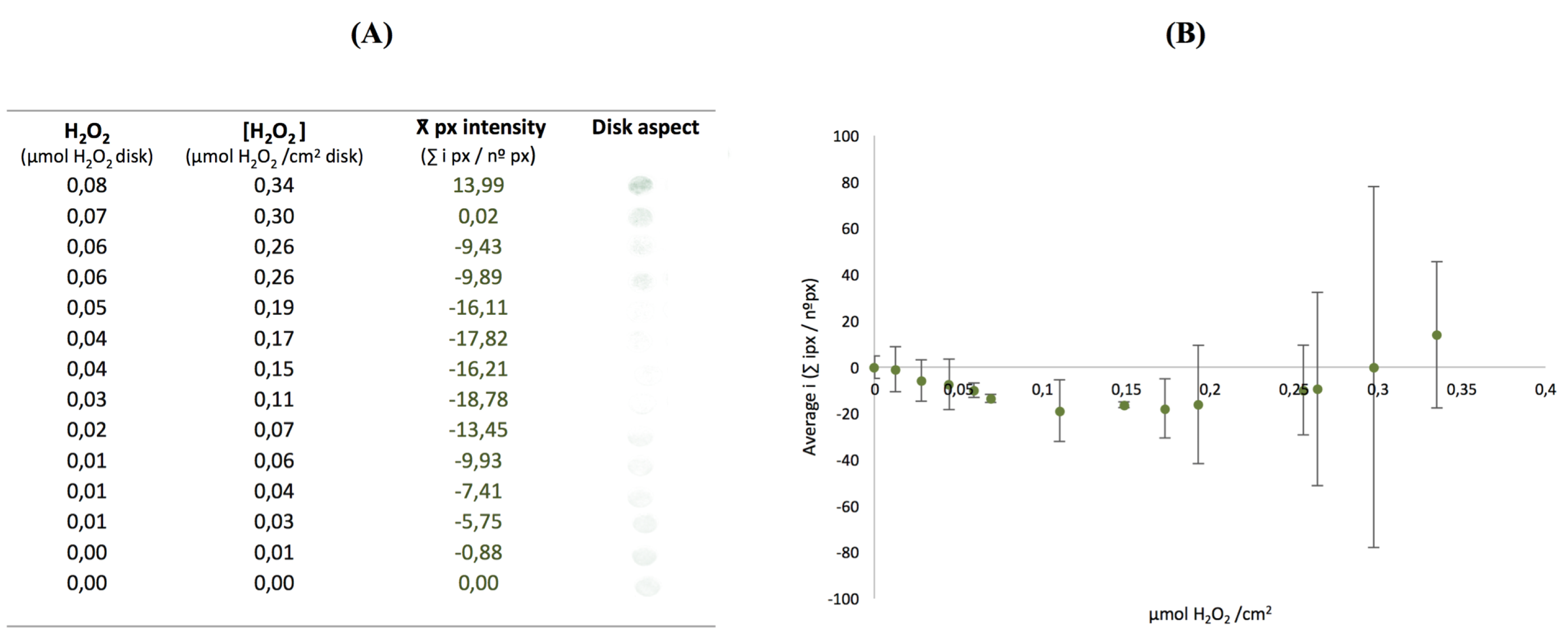

Supplement: FIGURE S1 — (A) Values of H2O2 concentration in the disks quantified in the complementary image, their average pixel intensity values (averages of three disk replicates) and aspect of the filter disks. (B) Relationship between average pixel intensity detected in the complimentary image and (±STDEV) and the amount of H2O2 per cm2 of disk. [file Image_1.tiff]
